# Detection of hepatic maturation by Raman spectroscopy in mesenchymal stromal cells undergoing hepatic differentiation

**DOI:** 10.1186/s13287-015-0259-y

**Published:** 2016-01-11

**Authors:** Hao-Hsiang Wu, Jennifer H. Ho, Oscar K. Lee

**Affiliations:** Institute of Biophotonics, National Yang-Ming University, No. 155, Sec.2, Linong Street, Taipei, 112 Taiwan; Center for Stem Cell Research, Wan Fang Hospital, Taipei Medical University, Taipei, 116 Taiwan; Graduate Institute of Clinical Medicine, Taipei Medical University, Taipei, Taiwan; Department of Ophthalmology, Wan Fang Hospital, Taipei Medical University, Taipei, Taiwan; Taipei City Hospital, No. 145, Zhengzhou Road, Datong District, Taipei, 10341 Taiwan; Institute of Clinical Medicine, National Yang-Ming University, Taipei, Taiwan; Stem Cell Research Center, National Yang-Ming University, Taipei, Taiwan; Department of Medical Research, Taipei Veterans General Hospital, Taipei, Taiwan; Department of Orthopaedics and Traumatology, Taipei Veterans General Hospital, Taipei, Taiwan

**Keywords:** Mesenchymal stromal cells, Hepatic differentiation, Raman spectroscopy

## Abstract

**Introduction:**

Mesenchymal stromal cells (MSCs) are well known for their application potential in tissue engineering. We previously reported that MSCs are able to differentiate into hepatocytes in vitro. However, conventional methods for estimating the maturation of hepatic differentiation require relatively large amounts of cell samples. Raman spectroscopy (RS), a photonic tool for acquisition of cell spectra by inelastic scattering, has been recently used as a label-free single-cell detector for biological applications including phenotypic changes and differentiation of cells and diagnosis. In this study, RS is used to real-time monitor the maturation of hepatic differentiation in live MSCs.

**Methods:**

The MSCs were cultured on the type I collagen pre-coating substrate and differentiated into hepatocytes in vitro using a two-step protocol. The Raman spectra at different time points are acquired in the range 400–3000 cm^–1^and analyzed by quantification methods and principle component analysis during hepatic differentiation from the MSCs.

**Results:**

The intensity of the broad band in the range 2800–3000 cm^–1^ reflects the amount of glycogen within lipochrome in differentiated hepatocytes. A high correlation coefficient between the glycogen amount and hepatic maturation was exhibited. Moreover, principle component analysis of the Raman spectra from 400 to 3000 cm^–1^ indicated that MSC-derived hepatocytes were close to the primary hepatocytes and were distinct from the undifferentiated MSCs.

**Conclusions:**

In summary, RS can serve as a rapid, non-invasive, real-time and label-free biosensor and reflects changes in live cell components during hepatic differentiation. The use of RS may thus facilitate the detection of hepatic differentiation and maturation in stem cells. Such an approach may substantially improve the feasibility as well as shorten the time required compared to the conventional molecular biology methods.

## Introduction

Inelastic scattering was first discovered by Raman [1]. When molecules are excited by incident laser light, the molecular vibration will emit scattered light. A small fraction of this scattered light has a different frequency from the incident laser light, approximately one of 10^7^ photons—a phenomenon called Raman scattering. The Raman shifts labeled as wave number units (cm^–1^) in the Raman spectra are the difference in wavelength between the scattered and incident light in the spectrum, and exhibit the characteristics of molecular vibration [2]. For a biological sample, the complex constituents (e.g., DNA, RNA, proteins, lipids and polysaccharides) in a cell generate a ‘molecular fingerprint’ on the Raman spectra [3]. In recent years, high resolution Raman spectroscopy (RS) has been rapidly utilized to provide an alternative tool for the detection of different cell/tissue types [4–8], or various cellular responses including apoptosis, necrosis and cell differentiation [9, 10]. Therefore, Raman spectra are able to provide unique information related to the molecular characteristics of live cells without labeling.

Stem cells are defined by their multidifferentiation potential and self-renewal capability. For tissue engineering, stem cells have provided a good means for growing new tissue and organs for transplantation [11, 12]. Mesenchymal stromal cells (MSCs) are adult stem cells which exhibit differentiation potential into bone, cartilage and adipose cells [13, 14]. They can also be isolated from various kinds of somatic tissues and culture-expanded easily in vitro. In our previous studies, we reported that MSCs were also successfully differentiated into hepatocyte-like cells in vitro [15]. Moreover, the therapeutic effect of MSC transplantation for liver failure in animal models suggests that MSCs may be useful for treating liver diseases [16–18]. However, estimating the maturation of differentiated cells before transplantation is a key challenge, and time-consuming conventional biological methods require the labeling or fixation of cells, making real-time monitoring impossible.

RS provides a faster and label-free biosensing approach to obtain cell spectra which show molecular vibrations for real-time measurement at the level of single live cells in different phenotypes. We reason that RS may be useful in detecting the stem cell commitment and differentiation. The purpose of the study is to evaluate the feasibility of using RS to measure the degree of maturation during hepatic differentiation of MSCs.

## Methods

### Isolation of murine MSCs and hepatocytes

Approval for these experiments was obtained from the Taipei Veterans General Hospital Institutional Animal Care and Use Committee (IACUC) regarding the use of animals prior to the commencement of the experiments. Murine MSCs were isolated from the bone marrow of 7–8-week-old Balb/c mice as previously described [19], and were cultured in low-glucose Dulbecco’s modified Eagle’s medium (LGDMEM; Sigma-Aldrich) supplemented with 10 % fetal bovine serum (FBS; Gibco) and 1 % PSG (Penicillin/Streptomycin/ Glutamine; Gibco). Primary murine hepatocytes were isolated from the liver of Balb/c mice euthanized by intraperitoneal injection of tribromoethanol (240 mg/kg) using a two-stage liver perfusion method reported in the literature [20, 21]. After perfusion, the liver was cut, the gall bladder was removed, and the liver was transferred to a new dish. The liver capsule was disrupted using tweezers, and the cells were released in a culture medium consisting of DMEM/F12 (Gibco) supplemented with 10 % FBS (Gibco) and 1 % PSG. The cell suspension was filtered using 70-μm strainers. The hepatocytes and nonparenchymal cells were separated by centrifugation at 50 g for 5 minutes. The hepatocytes pellet was then washed twice in the culture medium and the hepatocytes were cultured on type I collagen pre-coated cover glass.

### Hepatic differentiation of MSCs

MSCs were cultured on type I collagen (BD falcon) pre-coated cover glass, and then treated with step 1 of the hepatic differentiation medium consisting of Iscove’s modified Dulbecco’s medium (IMDM; Sigma-Aldrich) supplemented with 20 ng/mL hepatocyte growth factor, 10 ng/mL fibroblast growth factor-2, 0.61 g/L nicotinamide and 1 % PSG for the first week. Then the culture condition was changed for step 2 treatment with the hepatic differentiation medium consisting of IMDM supplemented with 20 ng/mL oncostatin M, 50 mg/mL ITS^+^, 1 μmol/L dexamethasone and 1 % PSG.

### Quantitative polymerase chain reaction

The total RNA of the MSCs was collected and quantified at different time points of hepatic differentiation. The mRNA was reverse-transcribed to cDNA by MMLV reverse transcriptase (EPICENTRE® Biotechnologies). The 10 ng cDNA in the total volume was analyzed by quantitative polymerase chain reaction (PCR).

### Albumin immunostaining

The cells were fixed using 3.7 % formaldehyde for 10 minutes, and permeablized with 0.1 % triton X-100 for 10 minutes. The cells were incubated with the primary antibody (R&D MAB1455) overnight at 4 °C and then incubated with secondary antibody (abcam ab150113) for 1 hour and washed with phosphate-buffered saline (PBS).

### Periodic acid–Schiff staining

The cells were fixed using 3.7 % formaldehyde for 10 minutes, and washed with PBS three times. The cells were then permeablized using 0.1 % triton X-100 for 10 minutes. Subsequently, the cells were treated with 1 % periodic acid for 5 minutes, and washed with H_2_O. Finally, they were treated with Schiff’s fuchsin-sulfite solution for 1 hour, washed lightly with H_2_O and dried.

### Glycogen storage assay

For quantitation of glycogen storage, the cells were lysed with H_2_O, and boiled for 10 minutes. The extract was used to quantify the glycogen using the Glycogen Colorimetric/Fluorometric Assay Kit (BioVision).

### Urea production assay

The urea production of MSCs after 3 weeks of hepatic differentiation was investigated. Cytoplasmic protein was extracted to analyze the urea production using a Urea Colorimetric Assay Kit (BioVision).

### Diastase treatment

The cells were fixed using 3.7 % formaldehyde for 10 minutes, and washed with PBS three times. The cells were then permeablized using 0.1 % triton X-100 for 10 minutes and washed by PBS. Subsequently, they were treated with 1 mg/ml diastase (Sigma) at 37 °C for 1 hour and washed by PBS.

### Raman spectroscopy and data acquisition

A Labram HR 800 Raman spectroscope (HORIBA Scientific, Japan) used He-Ne laser to excite the samples at 632.8 nm with 17 mW power. The spectroscope was equipped with an Olympus BX40, a 60× water immersion M-Plan objective (NA = 0.9), and a liquid nitrogen-cooled CCD two-dimensional array detector. The cells were seeded on cover glasses pre-coated with type I collagen, and the cover glasses were placed on top of mounting glasses with cells facing upwards. Then IMDM media were dropped on the cover glasses for the purpose of detection by a 60× water immersion M-Plan objective of Raman spectroscopy. Then the position for laser excitation in the monitor was chosen randomly for detecting, and the Raman spectra were acquired in the Raman shift range 400–3000 cm^–1^ with an integration time of 30 seconds. Cell number for data acquisition is over 10 in every measurement. The raw spectra data were nonlinearly smoothed, baseline corrected, and analyzed using LabSpec software.

### Principal component analysis

The Raman spectra (400–3000 cm^–1^) of the primary hepatocytes, undifferentiated MSCs, and MSC-derived hepatocytes were further processed by principal component analysis (PCA) using the Raman Processing (RB) program written in MATLAB developed by the SSIM/CARES research group at Wayne State University [22].

### Statistical analysis

The quantitative data were expressed as mean ± standard deviation. Statistical significance was determined by *t* test (*p* =0.05) or one-way analysis of variance with Tukey’s post-hoc test (*p* = 0.05) and different alphabetical letters indicate different levels of statistical significance at 95 % confidence intervals.

## Results and discussion

### *In vitro* hepatic differentiation of MSCs

**Fig. 1:**
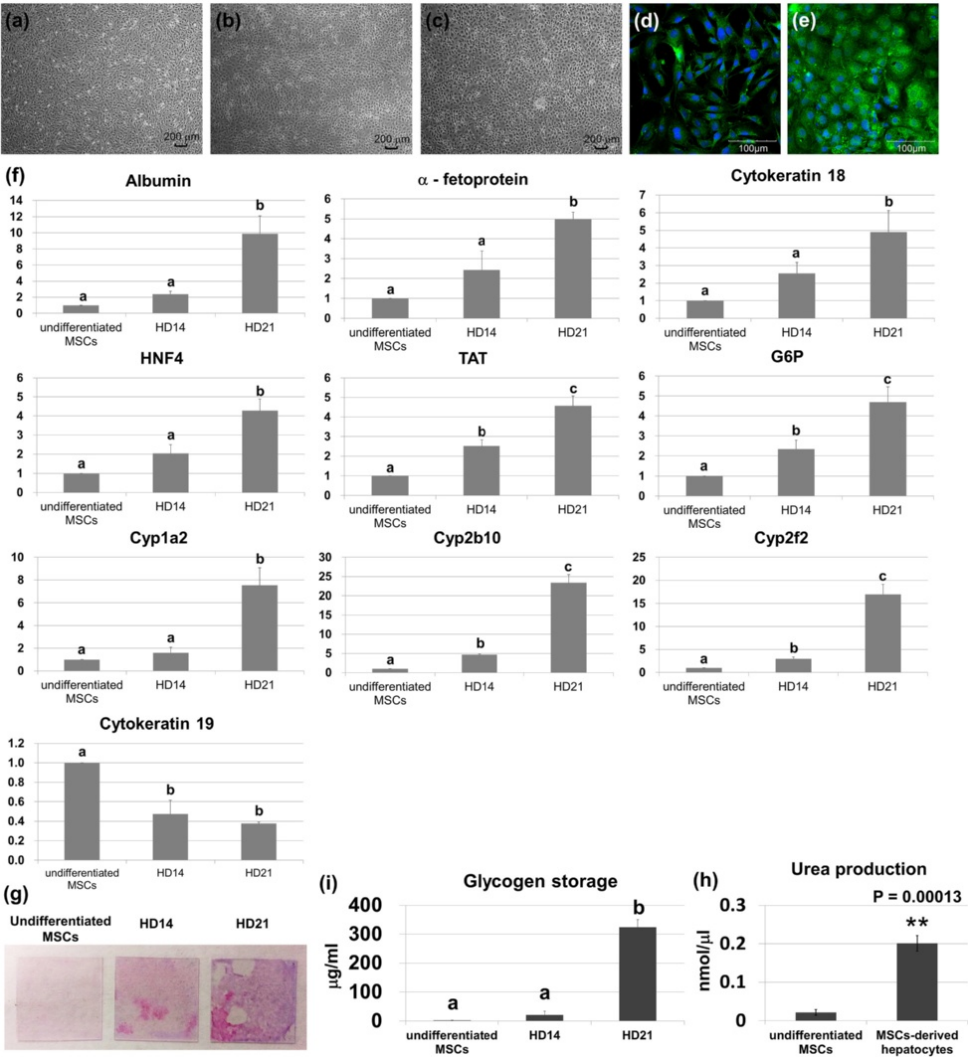
Hepatic differentiation of MSCs on cover glasses precoated with type I collagen. The MSCs were cultured on cover glasses pre-coated with type I collagen, and differentiated into hepatocytes. Cell morphology of **a** undifferentiated MSCs, **b** hepatic differentiation at day 14 and **c** hepatic differentiation at day 21. Albumin staining of **d** undifferentiated MSCs and **e** differentiated cells. **f** The mRNA expression of MSC-derived hepatocytes. The mRNA expression of liver-specific genes was investigated on days 14 (*HD14*) and 21 (*HD21*) of hepatic differentiation as well as in undifferentiated MSCs. **g** PAS staining of the undifferentiated MSCs, and at days 14 and 21 of hepatic differentiation. Quantification of **h** glycogen storage and **i** urea production during hepatic differentiation from the MSCs. Groups with different alphabetical letters are significantly different, whereas groups with the same alphabetical letters are not; *p < 0.05, **p < 0.01. *G6P* Glucose 6 phosphatase, *HNF4* Hepatic nuclear factor 4, *MSC* Mesenchymal stromal cell, *TAT* Tyrosine aminotransferase

To test the efficiency of hepatic differentiation of MSCs, cells were cultured on type I collagen coated cover glass and were induced into hepatocytes using a previously reported protocol [15]. The cells changed in shape from fibroblast-like to cuboidal, and then became hepatocyte-like in appearance (Fig. 1a, b, and c). Albumin, the most abundant blood plasma protein secreted by hepatocytes, was measured in the MSC-derived hepatocytes using immune-staining. The results indicated that albumin expression apparently increased 3 weeks after hepatic induction (Fig. 1d and e). The mRNA expression levels of liver-specific genes investigated by quantitative PCR revealed that albumin, α-fetoprotein, cytokeratin 18, hepatic nuclear factor 4, tyrosine aminotransferase, glucose 6 phosphatase, and metabolism-related cytochrome P450 enzymes (Cyp1a2, Cyp2b10, and Cyp2f2) were highly upregulated in the MSCs after hepatic induction, while cytokeratin 19 as a cholangiocyte marker was downregulated (Fig. 1f). In addition, the functional assays including glycogen storage and urea production were further investigated in the MSC-derived hepatocytes. Periodic acid–Schiff (PAS) staining and quantitative assay showed that, compared to undifferentiated MSCs, glycogen storage in the MSC-derived hepatocytes was significantly increased (Fig. 1g and h). Urea production was also elevated in the MSC-derived hepatocytes (Fig. 1i). These results indicated that the MSCs were efficiently differentiated into hepatocytes.

### Raman spectra of lipochrome in the MSC-derived hepatocytes

**Fig. 2:**
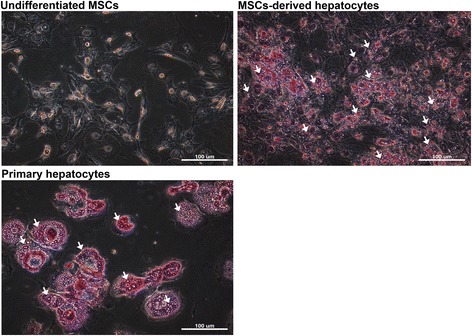
The lipochrome formation in cytoplasm increased in the MSC-derived hepatocytes and primary hepatocytes. The undifferentiated MSCs, MSC-derived hepatocytes and primary hepatocytes were stained with PAS staining. The *white arrows* indicate the PAS staining-positive granules

**Fig. 3:**
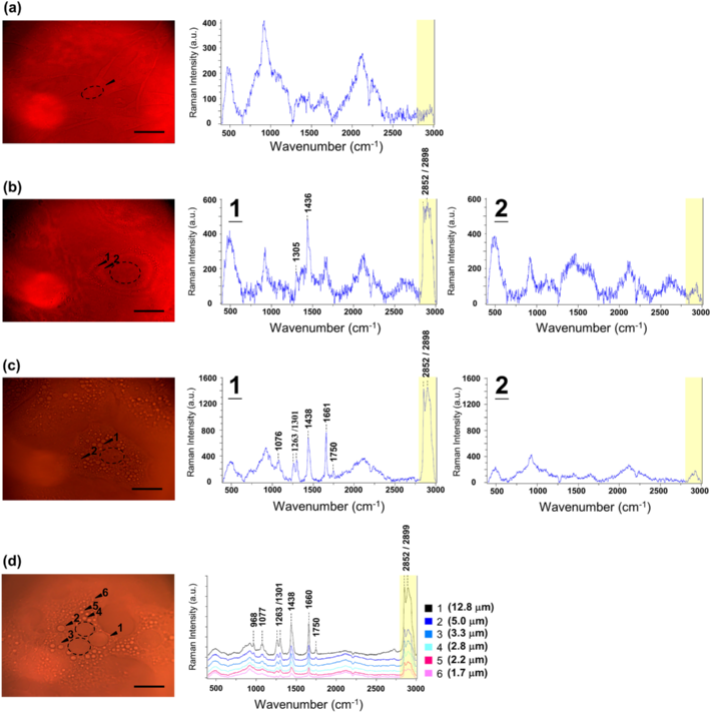
Raman spectra of lipochromes in the cytoplasm of the MSC-derived hepatocytes. The Raman spectra of **a** undifferentiated MSCs, **b** MSC-derived hepatocytes (1, lipochrome; 2, nonlipochrome cytoplasm), and **c** primary hepatocytes (1, lipochrome; 2, nonlipochrome cytoplasm) were investigated in 400–3000 cm^–1^. **d** Raman spectra of the lipochromes with different sizes (1-6) in the cytoplasm of the primary hepatocytes. The number in parentheses indicates the diameter of lipochromes. The *black arrow*s indicate the position of laser excitation and the *circle* with the dotted line indicates the nucleus. Scale bars = 20 μm

During hepatic differentiation, an increase in PAS staining-positive granules in the cytoplasm of MSC-derived cells was noted (Fig. 2). PAS staining is used to detect polysaccharides such as glycogen; also, glycoproteins, glycolipids, lipids, and phospholipids are positive under PAS staining. In hepatocytes, the granules are termed lipochrome (lipofuscin) which is PAS staining-positive and composed of lipid-containing residues of lysosomal digestion. Although lipids and proteins are the main constituents in lipochrome, a small amount of carbohydrate (4–7 %) is also found in lipochrome content [23], and glycogen is the major form of carbohydrate stored in hepatocytes. In rat liver, more than 10 % of cellular glycogen is located within lysosome [24]. Normally, the mean size of lipochrome is 1 μm, but larger granules are also encountered [25]. Moreover, the increased formation and size of lipochrome is reported to be associated with aging [26–29]. However, lipochrome is frequently found in postmitotic cells [30] and is also found in newborn human liver [25]. The maturation of hepatocytes was reported by in vitro hepatic induction of hepatic progenitor cells from fetal mouse liver, which showed increased PAS staining-positive granules along with induction time [31]. The above evidence indicates that it is possible to use lipochrome formation as an index of maturation level during hepatic differentiation of MSCs. To investigate Raman spectra between the MSC-derived hepatocytes and primary hepatocytes, the spectra were recorded in the range 400–3000 cm^–1^ and the assignment of the Raman shifts highlighted in Fig. 3 are summarized in Table 1 [32–35]. The spectra of lipochrome and nonlipochrome cytoplasmic position were investigated in the MSC-derived hepatocytes and primary hepatocytes. The lipochrome was not observed in the undifferentiated MSCs (Fig. 3a). Lipochrome spectra in the MSC-derived hepatocytes and primary hepatocytes both exhibited a broad band in the range 2800–3000 cm^–1^ with two main peaks (2852/2898 cm^–1^) indicating a CH_2_ symmetric stretch/CH stretch (Fig. 3b and c). In addition to the broad band, the MSC-derived hepatocytes and primary hepatocytes also have peaks of 1301–1305 cm^–1^ (C-H vibration, CH_2_ deformation, CH_3_ and CH_2_ twisting), 1436–1439 cm^–1^ (CH_2_ scissoring and CH_2_ deformation), and 1655–1680 cm^–1^ (Amide I) in the lipochrome spectra. However, the peaks at 1076 cm^–1^ for υ(C-C) or υ(C-O), 1263 cm^–1^for triglycerides, and 1750 cm^–1^ for υ(C = C) were only found in the lipochrome of the primary hepatocytes (Fig. 3c). In the nonlipochrome cytoplasmic position, both the MSC-derived hepatocytes and primary hepatocytes expressed a broad band in the range 2800–3000 cm^–1^ indicated as ν(C-H) as compared with the undifferentiated MSCs in Fig. 3. In the primary hepatocytes, the spectra intensity of the different sized lipochromes was found to increase with lipochrome size (Fig. 3d). Also, lipochromes of large size were observed in the primary hepatocytes, which are harvested from adult mice. Therefore, the lipochrome formation was found during hepatic differentiation from MSCs, and the vibration of the chemical bonding shown in the Raman spectrum of lipochrome could use for estimating maturation during hepatic differentiation.

**Table 1:** Assignment of the Raman shifts

Raman shift	Assignments
968 cm^–1^	Lipids
1076–1078 cm^–1^	υ(C-C) or υ(C-O)
1263–1264/1301–1303 cm^–1^	Triglycerides (fatty acids), =C-H/C-H vibration, CH_2_ deformation, CH_3_ and CH_2_ twisting
1436–1439 cm^–1^	CH_2_ scissoring, CH_2_ deformation
1655–1680 cm^–1^	Amide I
1750 cm^–1^	υ(C = C)
2852–2881/2899–2907 cm^–1^	CH_2_ symmetric stretch/CH stretch (lipids)
2800–3000 cm^–1^	υ(C − Η)

### Real-time monitoring of the maturation of MSC-derived hepatocytes by RS

**Fig. 4:**
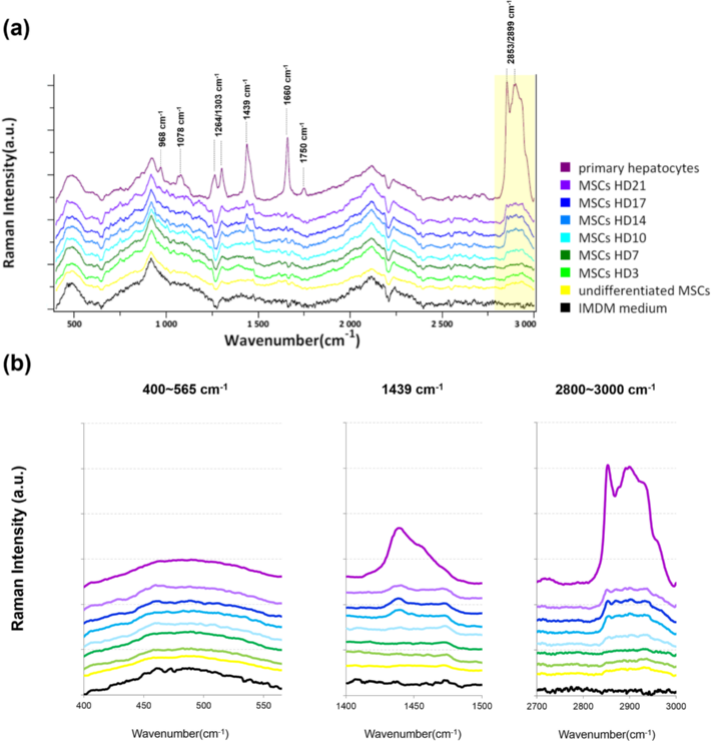
Real-time monitoring of hepatic differentiation from the MSCs by Raman spectroscopy. **a** Raman spectra in 400–3000 cm^–1^ was detected during hepatic differentiation from the MSCs. **b** The broad band ranges 400–565 cm^–1^ and 2800–3000 cm^–1^, and the peak at 1439 cm^–1^. *HD* Hepatic differentiation day, *MSC* Mesenchymal stromal cell

Time-dependent Raman spectra were measured to monitor the maturation of hepatic differentiation from the MSCs (Fig. 4a). The Raman spectra of primary hepatocytes were used as a reference and exhibited peaks at 968 cm^–1^, 1078 cm^–1^, 1264 cm^–1^, 1303 cm^–1^, 1439 cm^–1^, 1660 cm^–1^, and 1750 cm^–1^, and a broad band at 2800–3000 cm^–1^ composed of peaks at 2853 cm^–1^ and 2899 cm^–1^. Figure 4b magnifies the ranges at 400–565 cm^–1^, 1400–1500 cm^–1^, and 2700–3000 cm^–1^. The broad band intensity in the range 400–565 cm^–1^ was constant in the IMDM medium, undifferentiated MSCs, primary hepatocytes, and during the hepatocyte differentiation, indicating that signals in the range 400–565 cm^–1^ resulted from the background signal of the type I collagen coating the substrate. During the hepatic differentiation of the MSCs, the broad band intensity (2800–3000 cm^–1^) increased at day 10 and day 14 compared with the undifferentiated MSCs. In addition, a peak of 1439 cm^–1^ was detected at day 14 of the hepatic differentiation from the MSCs. However, the broad band intensity (2800–3000 cm^–1^) and peak (1439 cm^–1^) in the MSC-derived hepatocytes were lower than that for the primary hepatocytes. Because of the positive correlation between the broad band intensity (2800–3000 cm^–1^) and the duration of induction, we speculate that the signals at 2800–3000 cm^–1^ could be used to monitor the maturity of hepatic differentiation from the MSCs.

**Fig. 5:**
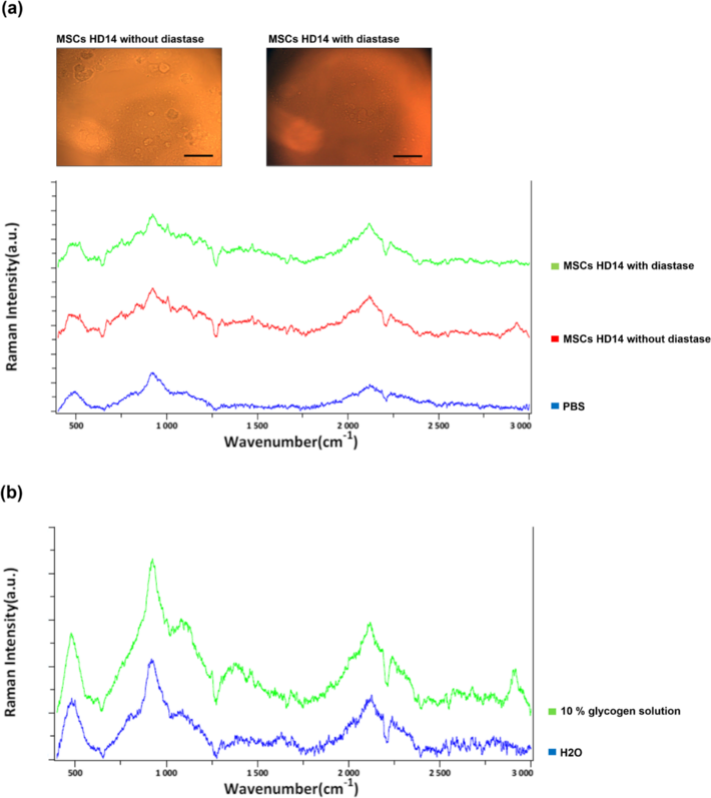
Raman spectra of the MSC-derived hepatocytes after glycogen digestion. **a** The cell morphology (*top*) and Raman spectra (*bottom*) of the MSC-derived hepatocytes at day 14 of hepatic differentiation with or without diastase treatment for glycogen catalysis. **b** The Raman spectra of 10 % glycogen solution. Scale bars = 20 μm. *HD* Hepatic differentiation day, *MSC* Mesenchymal stromal cell, *PBS* Phosphate-buffered saline

As shown in Fig. 2, lipochromes were positive for PAS staining in the MSC-derived hepatocytes. To further investigate which signal of the Raman spectra was from glycogen, MSC-derived hepatocytes were treated with diastase to catalyze the glycogen, and then the change in Raman signal was investigated. As shown in Fig. 5a, the broad band at 2800–3000 cm^–1^ in MSC-derived hepatocytes could no longer be detected after diastase treatment. Subsequently, to investigate whether amorphous glycogen also causes 2800–3000 cm^–1^ Raman shift, 10 % glycogen solution was tested. The result showed that the glycogen solution also caused Raman shift (2800–3000 cm^–1^) compared to H_2_O control (Fig. 5b). These results indicate that the signal was indeed derived from glycogen formation during hepatocyte differentiation.

### Quantification of maturation level during hepatic differentiation from the MSCs

**Fig. 6:**
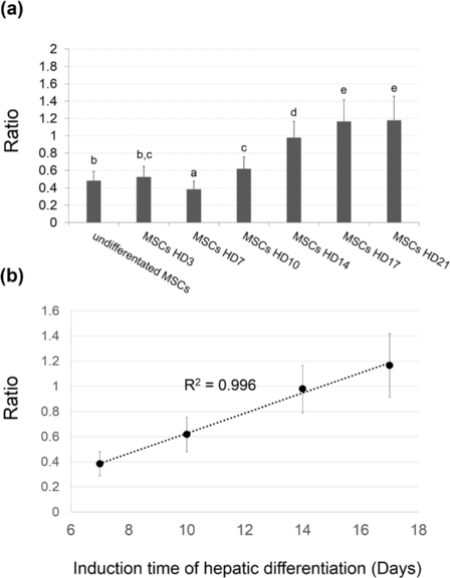
**a** Ratio of integral area (2800–3000 cm^–1^/400–565 cm^–1^) and **b** correlation of coefficient during hepatic differentiation of the MSCs. The ratio was increased and exhibited a high degree of correlation with the induction time from day 7 to day 17 of hepatic differentiation. Groups with different alphabetical letters are significantly different, whereas groups with same alphabetical letters are not. *HD* Hepatic differentiation day, *MSC* Mesenchymal stromal cell

To quantify the Raman spectra results, the integral area of the broad band (2800–3000 cm^–1^) was calculated and normalized according to the background signal of the broad band (400–565 cm^–1^) (Fig. 6a). At day 3 of hepatic differentiation, the ratio showed no significant difference as compared to the undifferentiated MSCs, but the ratio increased significantly at days 10, 14, 17, and 21. Furthermore, the ratio from day 7 to day 17 increased linearly and the correlation coefficient also exhibited a high degree of correlation with the induction time (R^2^ = 0.996) (Fig. 6b). The correlation coefficient from day 7 to day 17 indicated a high correlation between qualification of signals at 2800–3000 cm^–1^ and the maturation level during hepatic differentiation from the MSCs. Furthermore, it indicates gradual maturation in hepatic phenotype. The results indicate high correlation between the qualification of signals at 2800–3000 cm^–1^ and the maturation level during hepatic differentiation from the MSCs.

### PCA of Raman spectra in the MSC-derived hepatocytes

**Fig. 7:**
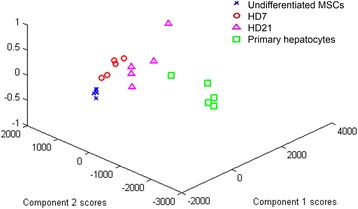
PCA of hepatic differentiation of the MSCs. The PCA exhibited different scores among the undifferentiated MSCs, MSCs under hepatic differentiation at day 7 and day 21, and primary hepatocytes. *HD* Hepatic differentiation day, *MSC* Mesenchymal stromal cell

To investigate the differences and similarities among the undifferentiated MSCs, MSC-derived hepatocytes, and primary hepatocytes, the Raman spectra were analyzed by PCA. As shown as Fig. 7, component scores of the undifferentiated MSCs and MSCs under hepatic differentiation for 7 and 21 days separated into three subgroups, indicating the different stages of hepatic differentiation. It was found that the MSC-derived hepatocytes at day 7 and day 21 were closer to the primary hepatocytes than the undifferentiated MSCs. The results indicate that the spectra of undifferentiated MSCs changed their Raman spectra signature to ones similar to the primary hepatocytes during hepatic differentiation.

A conventional molecular biology approach, such as quantitative PCR or immunostaining, to measure the maturation level of hepatic differentiation requires sacrificing a large number of cells, which makes real-time monitoring on live cells during hepatic differentiation impossible. In this study, we demonstrate that RS is a powerful tool for monitoring real-time molecular information at a single-cell level during hepatic differentiation from MSCs. The spectra of Raman scattering in MSC-derived hepatocytes during differentiation provide a unique fingerprint at each stage, which reflects the characteristics of molecular vibration composed of extremely complex cell components. RS can be therefore used for biosensing and to quantitatively evaluate the maturation level of hepatic differentiation from the MSCs by the signals at 2800–3000 cm^–1^.

The structure of multibranched polysaccharide in the glycogen structure contains a large amount of glycosidic bonds. Furthermore, Raman shift (2800–3000 cm^–1^) is also reported to represent the CH stretch from glyosidic linkage [36]. We show that the broad band (2800–3000 cm^–1^) assigned for ν(C-H) increased in intensity with hepatic differentiation (Fig. 4), indicating that the broad band signal is derived from glycosidic bonds of the glycogen structure in the MSC-derived hepatocytes by digesting glycogen (Fig. 5). Moreover, the real-time evaluation of the maturity level during hepatic differentiation of MSCs could be achieved by quantitative analysis of the Raman spectra at 2800–3000 cm^–1^ (Fig. 6), with the maturity measured by Raman spectra being comparable to that by conventional biomedical methods (Fig. 1).

During hepatic differentiation, lipochrome formation was also observed in the MSC-derived hepatocytes (Fig. 2). In this study, the spectra of the MSC-derived hepatocytes (Figs. 3 and 4) exhibit lower intensity in the broad band (2800–3000 cm^–1^) with two main peaks (2852/2898 cm^–1^) and one peak (1439 cm^–1^) indicating less lipid accumulation compared with that of the primary hepatocytes. However, primary hepatocytes exhibited the increased intensity of the 2852/2898 peaks along with the size of lipochrome (Figs. 3 and 4). It also indicates that lipids are accumulated in lipochromes of hepatocytes from adult mouse liver. One explanation is that the hepatocytes differentiated from MSCs under 21-day induction are functional, but are still relatively immature in comparison with the primary hepatocytes. Furthermore, three-dimensional scaffold structure composed of extracellular matrix support, other than induction of growth factors and cytokines, is a critical factor for hepatogenesis in liver development [37–42], but the three-dimensional scaffold is not present in this in vitro two-dimensional hepatic differentiation. Thus, the MSC-derived hepatocytes are not as mature as the primary hepatocytes in this study.

In this study, PCA of the Raman spectra for the undifferentiated MSCs and MSC-derived hepatocytes exhibited phenotypic difference on component score showing that PCA, in addition to signals at 2800–3000 cm^–1^, is a method to distinguish undifferentiated MSCs from MSC-derived hepatocytes (Fig. 7).

## Conclusion

In summary, RS can be used as a biosensor to provide real-time information on molecular characteristics during hepatocyte differentiation of MSCs. The findings have unequivocally demonstrated the usefulness of RS in stem cell studies. The application of RS may substantially facilitate stem cell research by overcoming the limitation of current molecular biological experimental methods.

## Abbreviations

DMEM: Dulbecco’s modified Eagle’s medium
FBS: Fetal bovine serum
IMDM: Iscove’s modified Dulbecco’s medium
MSC: Mesenchymal stromal cell
PAS: Periodic acid–Schiff
PBS: Phosphate-buffered saline
PCA: Principal component analysis
PCR: Polymerase chain reaction
PSG: Penicillin/Streptomycin/ Glutamine
RS: Raman spectroscopy

## References

[1] Raman CV (1928). A new radiation. Indian J Phys..

[2] Das RS, Agrawal YK (2011). Raman spectroscopy: recent advancements, techniques and applications. Vib Spectros.

[3] Chan JW, Lieu DK (2009). Label-free biochemical characterization of stem cells using vibrational spectroscopy. J Biophotonics.

[4] Hawi SR, Campbell WB, Kajdacsy-Balla A, Murphy R, Adar F, Nithipatikom K (1996). Characterization of normal and malignant human hepatocytes by Raman microspectroscopy. Cancer Lett.

[5] Huang Z, McWilliams A, Lam S, English J, McLean DI, Lui H (2003). Effect of formalin fixation on the near-infrared Raman spectroscopy of normal and cancerous human bronchial tissues. Int J Oncol.

[6] Galler K, Schleser F, Frohlich E, Requardt RP, Kortgen A, Bauer M (2014). Exploitation of the hepatic stellate cell Raman signature for their detection in native tissue samples. Integr Biol.

[7] Guo J, Cai W, Du B, Qian M, Sun Z (2009). Raman spectroscopic investigation on the interaction of malignant hepatocytes with doxorubicin. Biophys Chem.

[8] Majzner K, Kochan K, Kachamakova-Trojanowska N, Maslak E, Chlopicki S, Baranska M (2014). Raman imaging providing insights into chemical composition of lipid droplets of different size and origin: in hepatocytes and endothelium. Anal Chem.

[9] Brauchle E, Thude S, Brucker SY, Schenke-Layland K (2014). Cell death stages in single apoptotic and necrotic cells monitored by Raman microspectroscopy. Sci Rep..

[10] Hung PS, Kuo YC, Chen HG, Chiang HH, Lee OK (2013). Detection of osteogenic differentiation by differential mineralized matrix production in mesenchymal stromal cells by Raman spectroscopy. PLoS One.

[11] Atala A (2005). Tissue engineering, stem cells and cloning: current concepts and changing trends. Expert Opin Biol Ther.

[12] Griffith LG, Naughton G (2002). Tissue engineering--current challenges and expanding opportunities. Science (New York, NY).

[13] Tuan RS, Boland G, Tuli R (2003). Adult mesenchymal stem cells and cell-based tissue engineering. Arthritis Res Ther.

[14] Wang Y, Chen X, Cao W, Shi Y (2014). Plasticity of mesenchymal stem cells in immunomodulation: pathological and therapeutic implications. Nat Immunol.

[15] Lee KD, Kuo TK, Whang-Peng J, Chung YF, Lin CT, Chou SH (2004). In vitro hepatic differentiation of human mesenchymal stem cells. Hepatology (Baltimore, MD).

[16] Kuo TK, Hung SP, Chuang CH, Chen CT, Shih YR, Fang SC (2008). Stem cell therapy for liver disease: parameters governing the success of using bone marrow mesenchymal stem cells. Gastroenterology.

[17] Sun K, Xie X, Xie J, Jiao S, Chen X, Zhao X (2014). Cell-based therapy for acute and chronic liver failures: distinct diseases, different choices. Sci Rep..

[18] Volarevic V, Nurkovic J, Arsenijevic N, Stojkovic M (2014). Concise review: Therapeutic potential of mesenchymal stem cells for the treatment of acute liver failure and cirrhosis. Stem Cells (Dayton, OH).

[19] Peister A, Mellad JA, Larson BL, Hall BM, Gibson LF, Prockop DJ (2004). Adult stem cells from bone marrow (MSCs) isolated from different strains of inbred mice vary in surface epitopes, rates of proliferation, and differentiation potential. Blood.

[20] Klaunig JE, Goldblatt PJ, Hinton DE, Lipsky MM, Chacko J, Trump BF (1981). Mouse liver cell culture. I. Hepatocyte isolation. In Vitro.

[21] Klaunig JE, Goldblatt PJ, Hinton DE, Lipsky MM, Trump BF (1981). Mouse liver cell culture. II. Primary culture. In Vitro..

[22] Reisner LA, Cao A, Pandya AK (2011). An integrated software system for processing, analyzing, and classifying Raman spectra. Chemometr Intell Lab Syst.

[23] Brunk UT, Terman A (2002). Lipofuscin: mechanisms of age-related accumulation and influence on cell function12. Free Radic Biol Med.

[24] Geddes R, Stratton GC (1977). The influence of lysosomes on glycogen metabolism. Biochem J.

[25] Goldfischer S, Bernstein J (1969). Lipofuscin (aging) pigment granules of the newborn human liver. J Cell Biol.

[26] Höhn A, Jung T, Grimm S, Grune T (2010). Lipofuscin-bound iron is a major intracellular source of oxidants: role in senescent cells. Free Radic Biol Med.

[27] Koziolowa H, Dziezbicka E (1974). Advances in gastroenterology. Pigment deposits in the liver. I. Lipofuscins. Pol Arch Med Wewn.

[28] Tauchi H, Hananouchi M, Sato T (1980). Accumulation of lipofuscin pigment in human hepatic cells from different races and in different environmental conditions. Mech Ageing Dev.

[29] Ledda M, Barni L, Altieri L, Pannese E (1999). Amount and distribution of lipofuscin in nerve and satellite cells from spinal ganglia of young adult and aged rabbits. J Submicrosc Cytol Pathol.

[30] Terman A, Brunk UT (2004). Lipofuscin. Int J Biochem Cell Biol.

[31] Bi Y, He Y, Huang JY, Xu L, Tang N, He TC (2013). Induced maturation of hepatic progenitor cells *in vitro*. Braz J Med Biol Res.

[32] Galat A (1980). Study of the Raman scattering and infrared absorption spectra of branched polysaccharides. Acta Biochim Pol.

[33] Kizil R, Irudayaraj J, Seetharaman K (2002). Characterization of irradiated starches by using FT-Raman and FTIR spectroscopy. J Agric Food Chem.

[34] Movasaghi Z, Rehman S, Rehman IU (2007). Raman spectroscopy of biological tissues. Appl Spectros Rev.

[35] Aksoy C, Severcan F. Role of vibrational spectroscopy in stem cell research. Spectroscopy 2012;27(3). doi:10.1155/2012/513286.

[36] Abbate S, Conti G, Naggi A (1991). Characterisation of the glycosidic linkage by infrared and Raman spectroscopy in the C-H stretching region: α, α-trehalose and α, α-trehalose-2,3,4,6,6-d10. Carbohydr Res..

[37] Kazemnejad S (2009). Hepatic tissue engineering using scaffolds: state of the art. Avicenna Journal of Medical Biotechnology.

[38] Levenberg S, Huang NF, Lavik E, Rogers AB, Itskovitz-Eldor J, Langer R (2003). Differentiation of human embryonic stem cells on three-dimensional polymer scaffolds. Proc Natl Acad Sci U S A.

[39] Li J, Tao R, Wu W, Cao H, Xin J, Li J (2010). 3D PLGA scaffolds improve differentiation and function of bone marrow mesenchymal stem cell-derived hepatocytes. Stem Cells Dev.

[40] Liu T, Zhang S, Chen X, Li G, Wang Y (2010). Hepatic differentiation of mouse embryonic stem cells in three-dimensional polymer scaffolds. Tissue Eng Part A.

[41] Meng X, Leslie P, Zhang Y, Dong J (2014). Stem cells in a three-dimensional scaffold environment. SpringerPlus..

[42] Wu XB, Tao R (2012). Hepatocyte differentiation of mesenchymal stem cells. Hepatobiliary Pancreat Dis Int.

